# Subjective patient-reported versus objective adherence to subcutaneous interferon β-1a in multiple sclerosis using RebiSmart®: the CORE study

**DOI:** 10.1186/s12883-017-0952-9

**Published:** 2017-09-04

**Authors:** Chiara Zecca, Giulio Disanto, Sarah Mühl, Claudio Gobbi

**Affiliations:** 1 Multiple Sclerosis Center, Neurocenter of Southern Switzerland, Ospedale Regionale di Lugano, Lugano, Switzerland; 2 Merck (Schweiz) AG, Zug, Switzerland; 3 Multiple Sclerosis Center, Neurocenter of Southern Switzerland, Ospedale Regionale di Lugano, Via Tesserete 46, 6903 Lugano, Switzerland

**Keywords:** Multiple sclerosis, RebiSmart®, Autoinjector, Subcutaneous interferon β-1a, sc IFN β-1a, Adherence, Patient adherence, Self-reported adherence, Subjective adherence, Objective adherence

## Abstract

**Background:**

Patient adherence to treatment is key to preventing the worsening of neurological disability in multiple sclerosis (MS). The RebiSmart® autoinjector facilitates self-administration of subcutaneous interferon β-1a (sc IFN β-1a) and records objective adherence data. The CORE study was undertaken to evaluate the relationship between subjectively reported and objective adherence of MS patients using RebiSmart® in Switzerland and explore variables associated with objective adherence.

**Methods:**

Patients with relapsing-remitting MS who were treated with sc IFN β-1a 44 or 22 μg three times weekly using RebiSmart® for at least 9 months participated in this phase IV non-interventional study. Neurologist questionnaires were used at month 0 to collect patient demographics, medical history and estimates of patients’ adherence. Patient questionnaires were used to record subjective patient-reported adherence at month 0 and estimates of variables influencing adherence. Objective adherence data were obtained from the RebiSmart® log-files at months 0 and 6.

**Results:**

Of 56 patients who completed the observation period, 53 had evaluable data. Objective adherence differed significantly between self-reported compliant (*n* = 33) and non-compliant groups (*n* = 20) (*p* = 0.00001). Older age, greater disability, patient’s perception of the importance of ease of use and storage, being well informed about RebiSmart® features and neurologists’ estimations of adherence were all positively associated with treatment adherence.

**Conclusions:**

We showed for the first time that subjective patient-reported adherence in MS was well in line with objective adherence, suggesting that the frequency of administration is reported accurately by patients to their neurologist. This observation may have implications for future treatment monitoring strategies and strategic medical decisions. Patients, particularly those who are younger and with lower levels of disability, may benefit from being better informed of the importance of being adherent to their treatments and receiving information about their medication and the device they are using.

**Electronic supplementary material:**

The online version of this article (10.1186/s12883-017-0952-9) contains supplementary material, which is available to authorized users.

## Background

The importance of patient adherence to therapy for the effective management of chronic disease is well documented [1]. Poor adherence to treatment in chronic diseases may be exacerbated due to the need for treatment over the long-term. In some cases, the absence of symptoms over a period of time may be a reason for not adhering to, or even discontinuing, treatment. In other cases, progressive disability during the course of disease may lead to a perception of lack of treatment efficacy or increase difficulty or discomfort when self-administering treatment [2].

In multiple sclerosis (MS), patient adherence is a key factor to ensure improved clinical outcomes and has been associated with a reduced risk of relapse [3, 4], disability progression, hospitalization, MS-related medical costs [4, 5], and an improved quality of life [6]. Obtaining good adherence rates among patients is challenging, with rates as low as 30–40% observed in a retrospective study of patients with MS 2 years after initiating treatment with disease-modifying therapy [7].

Although reliable data are limited, evidence supports the use of tailored intervention strategies to improve patient adherence to therapy [8]. RebiSmart® (Merck Serono S.A., Geneva, Switzerland) is an electronic, multidose, autoinjector for subcutaneous (sc) self-injection of interferon (IFN) β-1a. RebiSmart® was designed to facilitate and increase the comfort level of self-injection for patients by manipulation of various comfort settings. The electronic log-file provides read-outs of objective adherence data, providing patients and physicians with reliable information about levels of compliance [9]. Thus, non-compliance can be easily detected and drive alternative treatment decisions.

The aims of this study were therefore to compare for the first time subjective patient-reported adherence with objectively recorded dosing history using RebiSmart® and to identify factors influencing MS therapy adherence in a Swiss population.

## Methods

CORE was a Swiss, practice survey-based study involving 11 centers. Patients with relapsing remitting MS (RRMS) who had received sc IFN β-1a 44 or 22 μg three times weekly (tiw) using RebiSmart® for at least 9 months, and regularly self-injecting the therapy, were included. They were treated according to the physician’s evaluation and decision.

All patients provided written informed consent and the responsible ethics committees were notified before study start.

Neurologist questionnaires (Additional file 1: Appendix I) were used to collect patient demographics and medical history at month 0 (M0). Patient questionnaires (Additional file 1: Appendix II) were used to record subjective adherence at M0 and after a 6-month observational period (M6). Patients reporting that they did not miss any injection during the study were defined as “self-reported adherent”; all remaining patients were “self-reported non-adherent”. Objective adherence data were collected from RebiSmart® device log-files for the 9-month period preceding M0 (retrospective adherence) and then between M0 and M6 (prospective adherence).

Objective adherence was calculated as the percentage of scheduled injections completed for each patient. Patients were grouped into three categories based on objective adherence data at M0: low (< 90%), medium (90–99.9%) and high (100%).

Objective adherence in self-reported adherent patients was compared to that in non-adherent patients using the Mann-Whitney U test (primary aim). As a secondary aim, retrospective and prospective objective adherence were compared using the Wilcoxon matched paired test. Furthermore, variables potentially associated with a greater objective adherence at M0 were explored using ordinal regression with low versus medium versus high objective adherence groups as the predicted variable.

Adverse drug reactions (ADRs) were reported by the treating physician and MS nurses.

## Results

Of 56 patients who completed the observation period, data were available for 53 (non-evaluable patient questionnaires, *n* = 2; treatment for < 9 months, *n* = 1). Patients had a median age of 49 years and most (77.4%) were female. At baseline, the median duration of treatment with sc IFN β-1a prior to study enrolment was 24 months (Table 1).

**Table 1:** Patient characteristics at baseline, *N* = 53

Patient characteristic	Median (IQR) or n (%)
Age, years	49.0 (38.0–55.0)
Female, n	41.0 (77.4%)
Last known EDSS score^a^	2.0 (1.5–3.3)
Time since diagnosis, years^b^	6.5 (3.0–12.0)
Duration of therapy, months^c^	47.0 (30.8–96.3)
Duration of therapy with sc IFN β-1a, months	24.0 (20.0–36.0)
Patients with relapse during past 9 months, n	8.0 (15.1%)

*EDSS* expanded disability status scale, *IFN* interferon, *IQR* interquartile range expressed as Q1–Q3, *sc* subcutaneous

^a^Data missing for 2 patients

^b^Data missing for 1 patient

^c^Data missing for 5 patients

Median objective adherence was significantly higher in self-reported adherent (100% [interquartile range, IQR: 98.8–100%], *n* = 33) than in self-reported non-adherent patients (93.4% [77.2–97.5%], *n* = 20) (*p* = 0.00001). There was no difference between retrospective (98.8% [IQR: 93–100%]) and prospective (98.8% [88.5–100%]) objective adherence (*p* = 0.75).

Fifteen (28.3%) patients had low objective adherence, 18 (34.0%) had medium adherence and 20 (37.7%) had high adherence at M0. Factors associated with significantly higher objective adherence were older age and greater expanded disability status scale (EDSS) score, neurologist’s subjective estimate of adherence, patient’s perceived importance of ease of use and ease of storage, and the patient feeling well informed about the features of RebiSmart®; previous MS therapy and perceived importance of treatment in delaying disease progression or importance of administration frequency were not associated (Table 2, Additional file 2). Fifteen cases of ADRs were reported in 11 patients, of which two were serious (depression and sarcoma; Additional file 3: Table S1).

**Table 2:** Factors impacting adherence

Factor	Objective adherence group			Ordinal regression		
	Low *n* = 15	Medium *n* = 18	High *n* = 20	OR	95% CI	p
Patient age, years	41.0 (31.5–48.0)	48.5 (36.5–54.8)	53.5 (42.0–63.0)	1.064	1.016–1.114	**0.008**
Last known EDSS score^a^	1.5 (1.0–2.0)	2.0 (1.5–3.3)	3.0 (2.0–3.8)	1.937	1.197–1.937	**0.008**
Neurologists’ estimations of adherence	10.0 (8.0–10.0)	9.0 (9.0–10.0)	10.0 (9.0–10.0)	1.528	1.019–2.291	**0.04**
Previous MS therapy	20.0%	33.0%	0.0%	0.344	0.089–1.340	0.124
Patient’s perceived relevance of ease of administration with RebiSmart®	9.0 (7.5–9.5)	10.0 (9.3–10.0)	10.0 (10.0–10.0)	1.578	1.080–2.307	**0.018**
Patient’s perceived relevance of storage of RebiSmart®	8.0 (5.5–8.0)	8.0 (5.3–10.0)	10.0 (8.0–10.0)	1.528	1.019–2.291	**0.04**
Being well informed about features of RebiSmart®	10.0 (9.0–10.0)	10.0 (10.0–10.0)	10.0 (10.0–10.0)	3.638	1.201–11.018	**0.022**
Patient’s perceived relevance of treatment in delaying progression of disease	10.0 (10.0–10.0)	10.0 (10.0–10.0)	10.0 (10.0–10.0)	1.063	0.644–1.753	0.812
Importance of frequency of administration	8.0 (7.0–9.0)	8.5 (6.3–10.0)	10.0 (5.0–10.0)	1.008	0.808–1.259	0.941

Data were analyzed by ordinal regression and are reported as median (interquartile range, Q1–Q3), with the exception of ‘Previous MS therapy’ which is reported as percentage of ‘yes’ responses. *P*-values that were considered significant (*p* < 0.05) are in bold type

Low adherence group: < 90%; medium adherence group: 90%–99.9%; high adherence group: > 99.9%

*CI* confidence interval, *EDSS* expanded disability status scale, *IFN* interferon, *OR* odds ratio, *sc* subcutaneous, *SD* standard deviation

^a^Data missing for 2 patients

## Discussion

In this Swiss MS population, objectively-measured adherence to sc IFN β-1a administered by RebiSmart® was very high, even with a median treatment duration of 2 years. These data were largely consistent with subjective self-reported adherence using questionnaires. Objective adherence was similarly high in the retrospective and prospective study periods, indicating overall good adherence levels before patients were included in the study, and thus suggesting that adherence was not greatly influenced by awareness of being monitored. These real-life adherence data are consistent with findings from other recent trials, in which similarly high levels of objective adherence have been reported for patients using RebiSmart®. In a large study conducted at various sites across Europe (SMART), mean objective adherence among 912 patients with RRMS, over a 12-month study period, was 97.1% [10]; another retrospective study in Italy of 114 RRMS patients using RebiSmart® and followed up during a 1.536 ± 0.961 year period reported a mean objective adherence of 95.0 ± 9.0% [11]; while a more recent long-term retrospective study in Spain of 110 patients with MS, reported median adherence of 96.5% for a median period of 2.7 years [12]; and a smaller study in Finland reported 93.5% objective adherence for 29 patients with RRMS followed up over 24 weeks [13]. The long-term RIVER study showed median overall objective adherence of 85.2% for 57 patients over a mean 20.5 month observational period [14].

Ordinal regression analysis indicated that adherence estimated by the treating neurologist was well in line with objective adherence, suggesting that the frequency of administration is reported accurately by patients to their neurologist and thus that the neurologists were generally aware of their patients’ adherence. This observation, together with our finding that objectively measured adherence was consistent with self-reported adherence, has implications in treatment decision making, i.e. supporting the need for switching disease modifying treatment or initiating strategies to improve patient compliance (such as frequent MS nurse monitoring) when poor adherence is suspected.

Ordinal regression analyses additionally showed that older age and greater disability were in this study associated with greater objective adherence to RebiSmart®. In another survey-based study of 708 patients in the U.S., patients who were older at diagnosis of MS were also found to have better adherence to treatment [15]. Furthermore, in a large prospective MS study in Australia, younger age at treatment initiation was predictive of discontinuation of disease-modifying therapy over a median follow-up period of 2 years [16]. However, patients with a higher EDSS score (greater disability) were also found to be more likely to discontinue treatment in the Australian study [16]. In a previous multicenter 12-month retrospective study of 384 RRMS patients in Italy, younger patients (≤ 25 years old) and those with EDSS score ≥ 4 at baseline again showed poorest adherence to sc IFN β-1a (79 and 71.4% of patients, respectively, with ≥ 80% completed doses), the most adherent patients being those aged 26–40 years at baseline and with EDSS score < 4 (over 90% of patients adherent) [17]. We hypothesize that older patients may have an increased awareness of the importance of treatment adherence for the prevention of further neurological disturbances. This may also be true for patients with greater disability in our study, despite the potential difficulties in performing self-injections in individuals with reduced physical capacity. The different findings observed for patients with greater disability in the Australian and Italian studies are likely due to differences in MS populations and study designs. Finally, patient perception of RebiSmart® ease of use and being well informed about the technical features of the device were also associated with higher adherence. A previous study in patients with other chronic disease (asthma and chronic obstructive pulmonary disease) have also reported the importance of being well informed about devices and medication for patients with chronic illness [18].

Limitations of the current study include selection bias towards compliant MS populations participating in the study, which is inherent to studies of this nature. Thus, these findings must be interpreted with caution. This was an exploratory study and the questionnaire will need further validation in additional cohorts of patients. The limited sample size may also have prevented the detection of additional factors potentially influencing adherence. Finally we chose adherence thresholds that were stricter than those usually reported [6, 19, 20], to allow a more equal distribution of patients across categories and a more valid statistical analysis. Indeed, only seven patients in this study had an objective adherence below 80% and this did not allow us to reliably investigate associations with adherence measures inferior to this threshold. While we cannot exclude the possibility that other factors could be associated with low adherence to injections, we believe our data can provide useful information for neurologists and care givers in MS. Our findings are also in-line with more recent publications concerning adherence to injectables at the observational study level [12, 13].

## Conclusions

Patient-reported adherence was substantially in line with objective adherence, indicating that the frequently of administration is reported accurately by patients to their neurologists. The adherence to injectable treatments in patients with MS using RebiSmart®was very high. The importance of being adherent to treatments should be particularly stressed in younger and less disabled individuals and all patients should be well informed about their medication and the device they are using.

## Additional files

Additional file 1: Neurologist and patient questionnaires. Neurologist questionnaire (Appendix I) used to collect patient demographics and medical history at month 0 and patient questionnaire (Appendix II) used to record subjective adherence at M0 and after a 6-month observational period. (PDF 73 kb)

Additional file 2: Series of six figures comparing adherence with age, EDSS score, neurologists’ estimations of adherence, ease of administration, patient’s perceived relevance of storage and being well informed about RebiSmart® features. (PDF 43 kb)

Additional file 3: Table S1. List of individual adverse drug reactions. Details of 15 cases of adverse drug reactions reported by 11 patients. (DOCX 14 kb)

## Abbreviations

ADRs: Adverse drug reactions
EDSS: Expanded disability status scale (EDSS)
IFN: Interferon (IFN)
IQR: Interquartile range (IQR)
M0: Month 0 (M0)
M6: 6-month observational period (M6)
MS: Multiple sclerosis (MS)
RMS: Relapsing remitting MS (RRMS)
sc IFN β-1a: Subcutaneous interferon β-1a (sc IFN β-1a)
sc: Subcutaneous (sc)
tiw: Three times weekly (tiw)
