# Supplementary material for: Subjective patient-reported versus objective adherence to subcutaneous interferon β-1a in multiple sclerosis using RebiSmart®: the CORE study
Source: BMC Neurol. 2017 Sep 4;17:171. doi: 10.1186/s12883-017-0952-9 (PMC5584024; doi:10.1186/s12883-017-0952-9)
Supplement: Supplementary file 1 — Neurologist and patient questionnaires. Neurologist questionnaire (Appendix I) used to collect patient demographics and medical history at month 0 and patient questionnaire (Appendix II) used to record subjective adherence at M0 and after a 6-month observational period. (PDF 73 kb) [file 12883_2017_952_MOESM1_ESM.pdf]

Appendix I – Questionnaire for the neurologist

**General Data**		
[Site number]: ☐	[Patient number]: ☐	
Gender of patient:	F ☐ / M ☐	
Date of birth: ☐		
Diagnosis of multiple sclerosis:	since ☐ year(s)	
MS-Treatment duration : ☐ Months	with RebiSmart: ☐ months	
Last known EDSS Scale: ☐	Date: ☐	
Relapses within the last 9 months:	☐	
Previous MS-therapy (if applicable):	☐	
Reasons for treatment change (if applicable):	☐	
Comorbidities (if applicable):	☐	
How often did you see the patient within the last 9 months?	☐ times	

Subjective Perception

On a scale from 1 to 10, in which 1=not at all adherent and 10=totally adherent, how would you rate the adherence of your patient to RebiSmart?

**1**									**10**

Appendix II – Patient questionnaire

**General Data**		
[Site number]: ☐	[Patient number]: ☐	
Gender of patient:	F ☐ /	M ☐
Date of birth: ☐		

a) Perception of the disease

Question 1:
How far do you think MS negatively impacts the quality of your life?
☐ Not at all ☐ Not very much ☐ Quite a lot ☐ A lot

b) Sources of information

Question 2:
How far do you, on your own initiative, seek out further information on MS and on other therapies for MS?
☐ Not at all ☐ Not very much ☐ Quite a lot ☐ A lot

If not at all, ☞go to question 5

***Question* 3:**
From the following list, which are the three most important means of information you are currently using, and from which you would not want to refrain from using? (from 1 to 3, in order of importance; 1 = the most important source of information)
☐ Neurologist (incl. booklets or leaflets in private practice)
☐ GP (incl. booklets or leaflets in private practice)
☐ Patients‘ organisation/ Multiple Sclerosis association
☐ Specialised magazines or books
☐ Pharmacist
☐ Nurse

☐ The internet, please specify which websites:
☐ Social Media (Facebook, Twitter ..), please specify:
☐ Other, please specify:

Question 4:
Did it already happen, that based on the information you have found, you have decided not to apply (anymore) your treatment with Rebif®? <br>Yes ☐ / No ☐

c) Therapy

Question 5:
How often did you have contact with your MS nurse within the last 9 months? times

***Question* 6:**
How much importance do you attach to the following aspects with regard to the therapy of the multiple sclerosis? On a scale from 1 to 10, in which 1= not at all important and 10=extremely important

	Importance (1=not at all important to 10=extremely important)
Efficacy in reducing the relapse rate	
Efficacy in delaying the progression of the disease	
Maintenance of the efficacy even after long period of treatment	
Lack of side effects	
Ease in administering the therapy	

Frequency of administration	
Storage of the therapy	

Question 7:

On a scale from 1 to 10, in which 1= do not agree at all and 10= totally agree, how much do you agree with the following statements regarding your treatment with Rebif®?

The treatment I'm currently using provides benefits

**1**									**10**

I'm afraid of possible side effects that may be associated with the therapy

**1**									**10**

I am well informed about the features of the RebiSmart

**1**									**10**

Since the first injection I feel confident with the injection procedure with RebiSmart

**1**									**10**

d) Satisfaction with the therapy

Question 8:

Using a score from 1 (not at all satisfied) to 10 (completely satisfied), how satisfied do you feel with your current therapy?

**1**									**10**

***Question* 9:**

More specifically, how do you rate the therapy that you are currently taking on the following aspects? On a scale from 1 to 10, in which 1= not at all satisfied and 10=completely satisfied

	Satisfaction (1=not at all satisfied to 10=completely satisfied)
Efficacy in reducing the relapse rate	
Efficacy in delaying the progression of the disease	
Maintenance of the efficacy even after long period of treatment	
Lack of side effects	
Ease in administering the therapy	
Frequency of administration	
Storage of the therapy	

Question 10:

On a scale from 1 to 10, in which 1= not easy at all and 10= very easy, how easy would you rate the handling of RebiSmart?

**1**									**10**

e) Frequence of the injection

Question 11:
Do you apply your treatment with Rebif® regularly? Yes ☐ / No ☐

If yes, ☞go to question 12a
If no, ☞go to question 12b

Question 12a:
How do you define regularly? (single answer)<br><br>☐ I have **never** missed one single application of my treatment with Rebif®<br><br>or over one month I forget to apply my treatment with Rebif®<br><br>☐**once** ☐**twice** ☐**thrice** ☐**four times or more**

Question 12b:
How do you define not regularly? (single answer)<br><br>Over one month I forget to apply my treatment with Rebif®<br><br>☐**once** ☐**twice** ☐**thrice** ☐**four times or more**

***Question* 13:**
How many Rebif®-injections have you missed within the last 9 months? (estimation)

☐ none
☐ one Rebif®-injection over 9 months
☐ one Rebif®-injection every 3 months – i.e. 3 injections
☐ one Rebif®-injection per month – i.e. 9 injections
☐ more than 9 Rebif®-injections

If none, ☞go to question 15

***Question* 14:**
On a scale from 1 to 10, in which 1=not at all important and 10=extremely important, how important are the following factors with regard to the missed Rebif®-injections?

Forgetfulness

**1**									**10**

Lack of motivation

**1**									**10**

Managing the number of injections in relation with your state of health (I feel good)

**1**									**10**

Disturbing factors associated with the injection

**1**									**10**

Fear of the injection

**1**									**10**

I feel good

**1**									**10**

Adverse drug reactions (please complete form attached → Appendix III)

**1**									**10**

Question 15:
Were there specific situations during which you did not inject your treatment with Rebif® within the last 9 months?<br>Yes ☐ / No ☐

If yes, ☞go to question 16
If no, ☞go to question 17

***Question* 16:**
Which of the following examples describe the most those specific situations where you did not inject your treatment with Rebif® within the last 9 months? (multiple choices)

☐During holidays
☐Lack of motivation
☐Issue with RebiSmart<br>If technical issue, already reported?<br>☐ Yes

☐ No (please complete form attached)
☐Other

Question 17:
Do you use any methods/tools to help you remember to apply your treatment with Rebif®? Yes ☐ / No ☐

If yes, ☞go to question 18
If no, ☞go to question 19

***Question* 18:**
Which of the following methods/ tools are you currently using to remind you to apply your treatment with Rebif®?
☐Reminder function of RebiSmart
☐Text messaging services
☐Application for Smartphone (iPhone, Android...)
☐Other, please specify:

***Question* 19:**
How often do you look at the dose history calendar in the RebiSmart device?
☐Never
☐Once in a week
☐Once in a month
☐Other, please specify:

f) <u>RebiSmart adherence read-out (via RebiSmart Download Software/iMed)</u>

***Question* 20:**
Average adherence over the last 9 months:
%
