# Supplementary figures and images for: Subjective patient-reported versus objective adherence to subcutaneous interferon β-1a in multiple sclerosis using RebiSmart®: the CORE study
Source: BMC Neurol. 2017 Sep 4;17:171. doi: 10.1186/s12883-017-0952-9 (PMC5584024; doi:10.1186/s12883-017-0952-9)

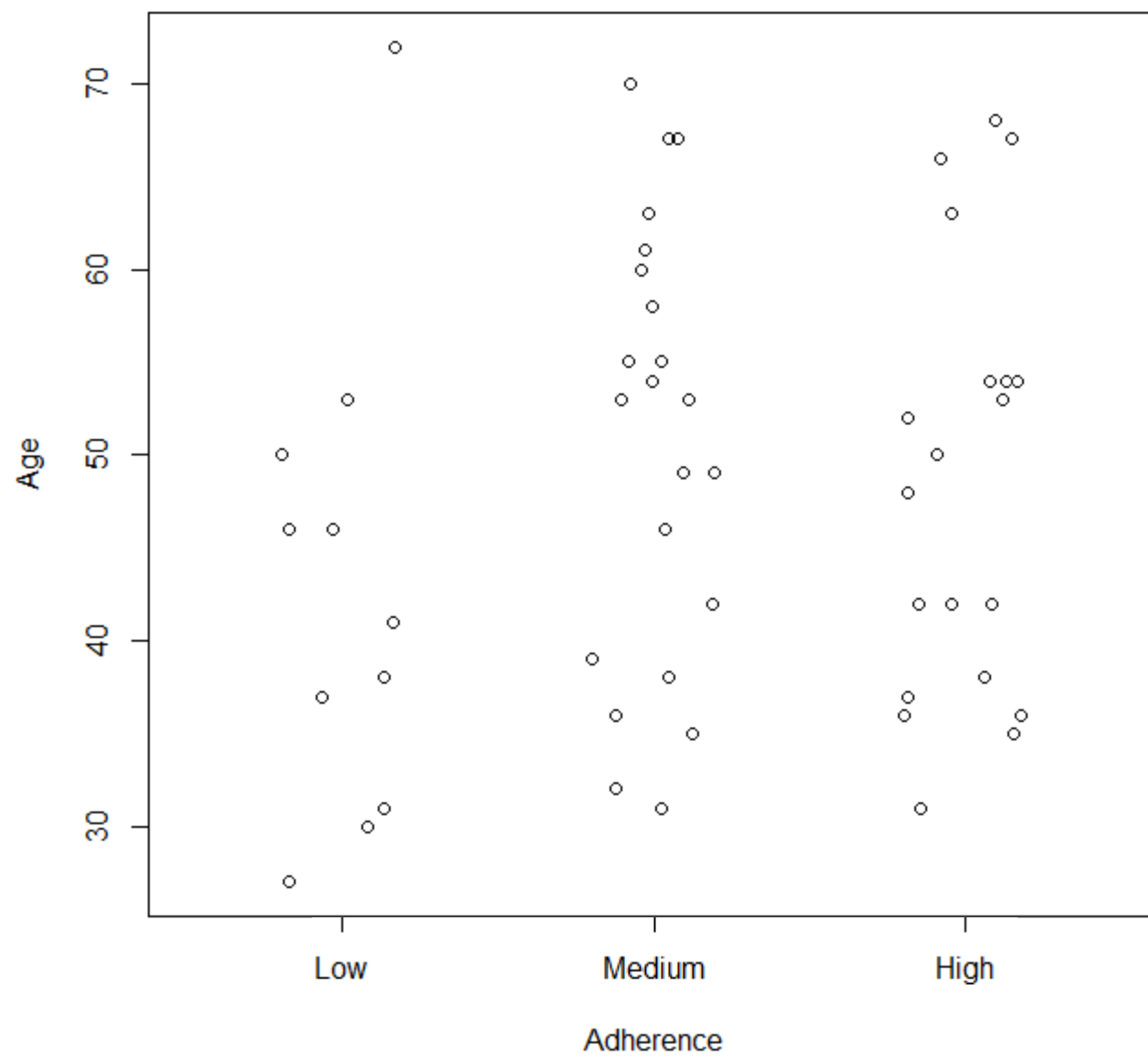

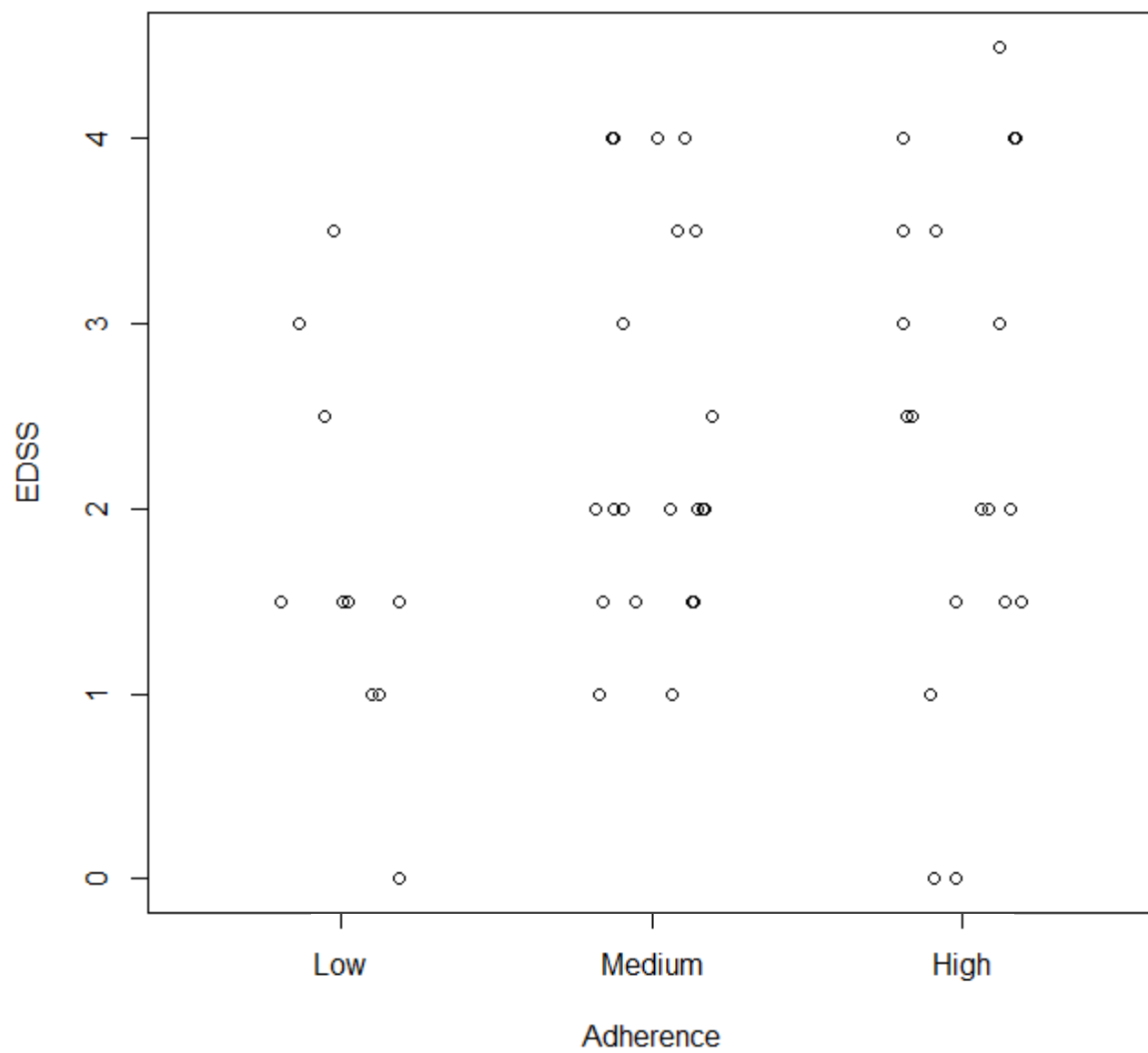

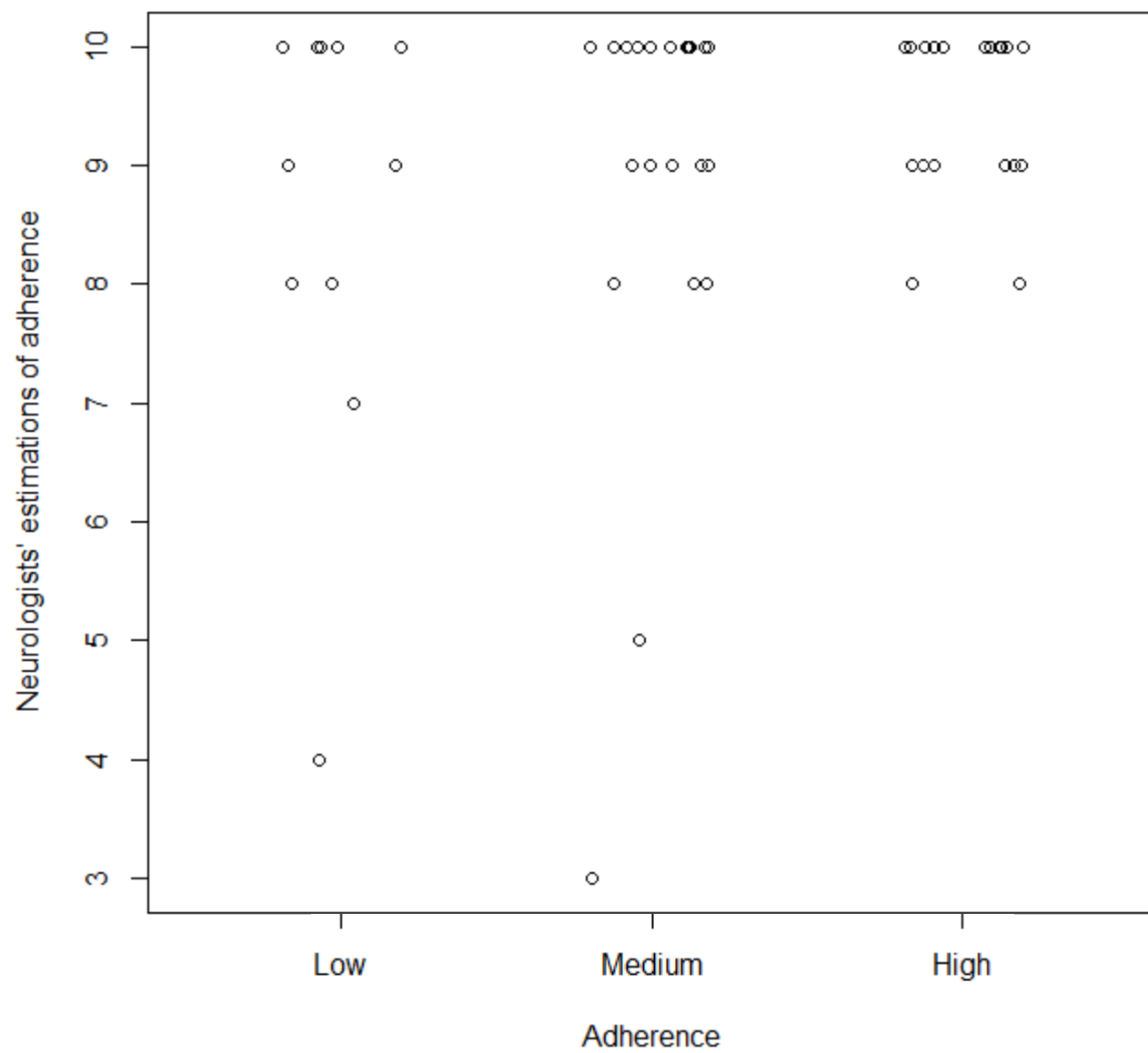

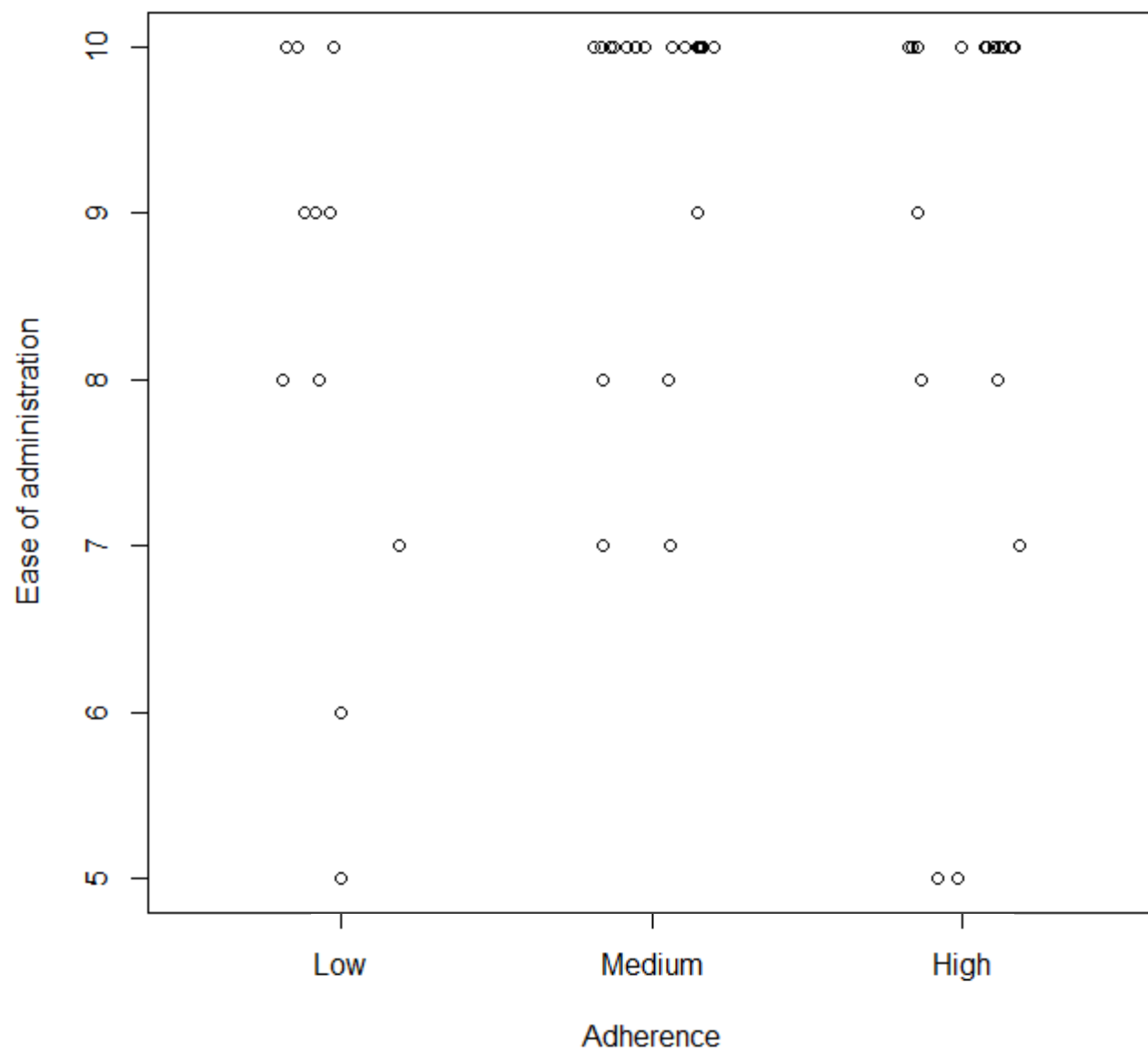

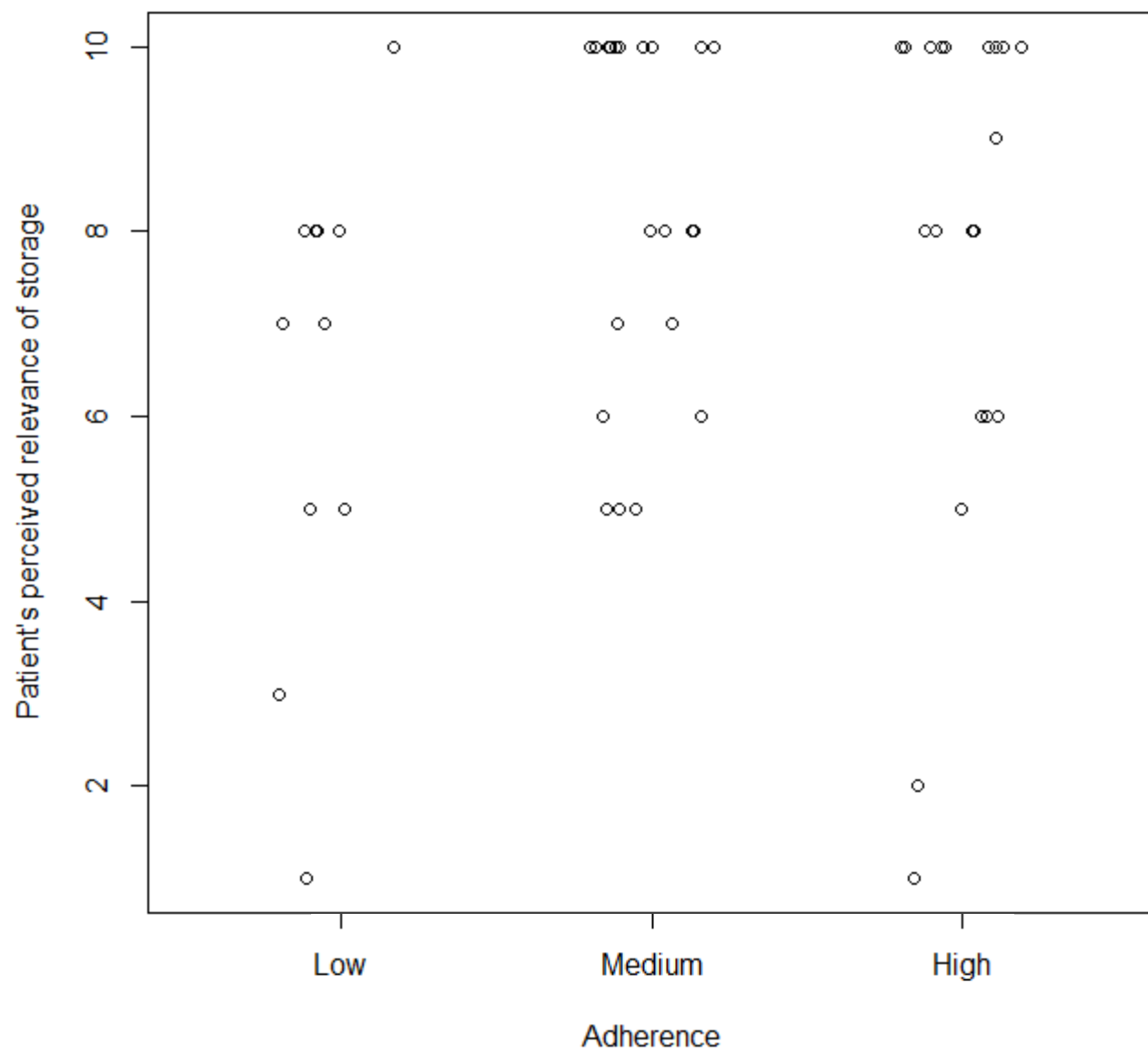

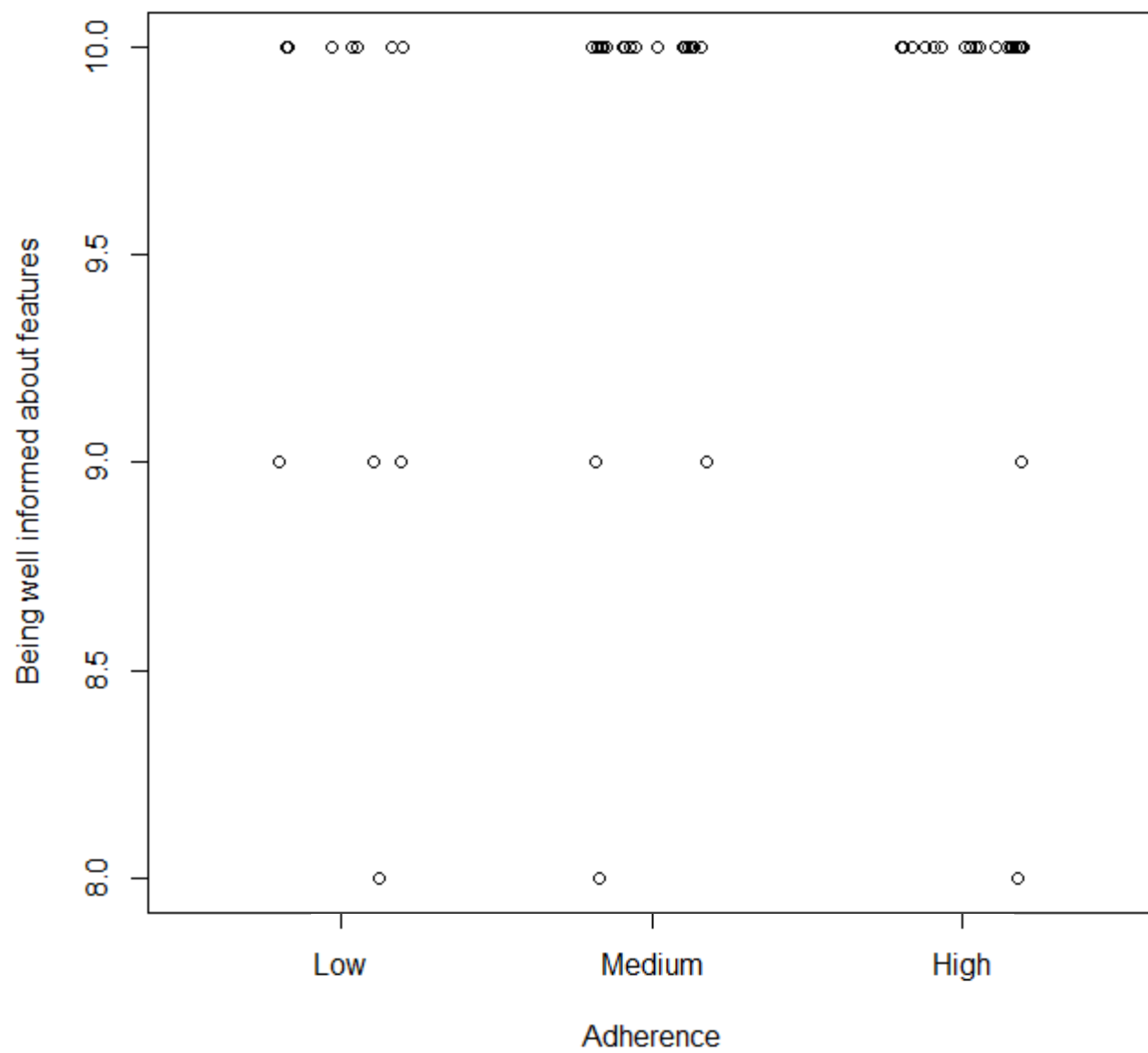

Supplement: Supplementary file 2 — Series of six figures comparing adherence with age, EDSS score, neurologists’ estimations of adherence, ease of administration, patient’s perceived relevance of storage and being well informed about RebiSmart® features. (PDF 43 kb) [file 12883_2017_952_MOESM2_ESM.pdf]
