# Supplementary material for: Subjective patient-reported versus objective adherence to subcutaneous interferon β-1a in multiple sclerosis using RebiSmart®: the CORE study
Source: BMC Neurol. 2017 Sep 4;17:171. doi: 10.1186/s12883-017-0952-9 (PMC5584024; doi:10.1186/s12883-017-0952-9)
Supplement: Supplementary file 3 — List of individual adverse drug reactions. Details of 15 cases of adverse drug reactions reported by 11 patients. (DOCX 14 kb) [file 12883_2017_952_MOESM3_ESM.docx]

**Supplementary Table S1** List of individual adverse drug reactions

| PATIENT | REPORTED TERM | SERIOUSNESS |
| --- | --- | --- |
| Patient 1 | Flu-like symptoms after injection, approximately 1–2 times per month | Not serious |
| Patient 2 | Flu-like symptoms | Not serious |
| Patient 2 | Redness of the skin | Not serious |
| Patient 3 | Flu-like symptoms | Not serious |
| Patient 3 | Spots at the injection site | Not serious |
| Patient 4 | Flu-like symptoms | Not serious |
| Patient 4 | Headache | Not serious |
| Patient 5 | Flu-like symptoms | Not serious |
| Patient 6 | Depressed mood | Not serious |
| Patient 7 | Flu-like symptoms | Not serious |
| Patient 7 | Injection site reactions | Not serious |
| Patient 8 | Pain in the thigh | Not serious |
| Patient 9 | Light skin redness | Not serious |
| Patient 10 | Depression | Serious |
| Patient 11 | Sarcoma | Serious |
